# Effect of repeated sessions of dual-site anodal tDCS on post-exercise performance and subjective recovery in recreationally active females: a randomized controlled trial

**DOI:** 10.3389/fphys.2025.1649732

**Published:** 2025-09-16

**Authors:** Shima Sharifi Melahbid, Ehsan Amiri, Vahid Tadibi, Daniel Gomes da Silva Machado

**Affiliations:** ^1^ Exercise Metabolism and Performance Lab (EMPL), Department of Exercise Physiology, Faculty of Sport Sciences, Razi University, Kermanshah, Iran; ^2^ Department of Sport Science, Human Performance Research Center (HPRC), University of Konstanz, Konstanz, Germany; ^3^ Department of Physical Education, Research Group in Neuroscience of Human Movement (NeuroMove), Federal University of Rio Grande do Norte, Natal, Brazil

**Keywords:** transcranial direct current stimulation (tDCS), neuromodulation, performance recovery, dual-site stimulation, endurance exercise, subjective recovery, motor cortex (M1), dorsolateral prefrontal cortex (DLPFC)

## Abstract

**Background:**

Previous studies have investigated the effects of transcranial direct current stimulation (tDCS) on performance enhancement, but limited research has examined its impact on post-exercise recovery. This study aimed to assess the effects of three consecutive sessions of dual-site anodal tDCS, targeting the primary motor cortex (M1) and the left dorsolateral prefrontal cortex (L-DLPFC), on both subjective and objective recovery measures in recreationally active females.

**Methods:**

Twenty-five recreationally active females were randomly assigned to either an anodal tDCS group (n = 13) or a sham group (n = 12). Performance and recovery were assessed at three time points: (1) before tDCS intervention, (2) immediately after a fatigue-inducing time-to-exhaustion test, and (3) following a 24-h recovery period. Participants completed a 3-km cycling time trial (TT) and a Sargent Jump Test (SJT) at each assessment. Additionally, after 24 h of recovery, they completed the Total Quality Recovery (TQR) scale and the Wellbeing Questionnaire (WBQ). Following baseline measurements, participants received their assigned intervention, three consecutive daily stimulation sessions (2 mA, 20 min, targeting + F3/-AF8 and +Cz/-AFz simultaneously), before the fatigue-inducing task.

**Results:**

Both groups exhibited similar physiological and perceived exertion responses during the fatigue-inducing task (all p > 0.05). While the a-tDCS group showed significant improvements in 3-km TT performance at 24 h post-recovery compared to baseline (p < 0.001, 95% CI [-36.71, −11.33]) and post-fatigue (p < 0.001, 95% CI [-28.4, −8.96]), there were no between-group differences (p > 0.05). However, the tDCS group reported higher TQR scores than the sham group at 24 h (p = 0.046, 95% CI [0.000, 2.000]). No significant between-group differences were observed in explosive performance (SJT) or WBQ scores (all p > 0.05).

**Conclusion:**

Three sessions of dual-site a-tDCS targeting M1 and L-DLPFC may enhance perceived recovery (TQR) in recreationally active females, but do not significantly influence wellbeing (WBQ) or objective performance recovery measures. The benefit appears to be subjective only, without a measurable performance advantage.

**Clinical trial registration:**

The trial was registered in the Iranian Clinical Trial Registry (www.irct.behdasht.gov.ir, IRCT ID: IRCT20230925059509N1).

## 1 Background

Effective recovery between training sessions or competitive events (e.g., soccer matches, combat sport rounds) is essential for restoring psychophysiological systems to optimal levels before subsequent performance demands (Kellmann et al., 2018). Recovery has become increasingly recognized as a critical component of athletic preparation, as an imbalance between training stimulus and recovery may lead to undesirable outcomes (Kellmann et al., 2018). These include non-functional overreaching (characterized by performance decrements without subsequent supercompensation, requiring weeks to resolve) or, in more severe cases, overtraining syndrome (marked by chronic performance decline and associated symptoms that may persist for months or years) (Giboin et al., 2018; Kargarfard et al., 2018; Baghaei et al., 2022; Bellinger, 2020).

Proper implementation and monitoring of recovery strategies serve two critical purposes: preventing negative training outcomes and optimizing the training adaptation process (Kellmann et al., 2018). Various legal recovery modalities have been employed for this purpose, including compression garments, cold-water immersion, cryotherapy, and massage (Li et al., 2024). However, the empirical evidence supporting their efficacy remains inconsistent (Li et al., 2024). Notably, most existing recovery strategies primarily target peripheral aspects of fatigue while overlooking the role of the central nervous system (CNS) in recovery processes (Rattray et al., 2015; Minett and Duffield, 2014). Emerging research demonstrates that CNS recovery may lag behind peripheral recovery. For instance, Latella et al. (2016) found that while muscle strength recovered within 6 h after heavy strength training, corticospinal excitability, a neurophysiological parameter related to muscle control, required 48 h to recover. This suggests the CNS may compensate for reduced excitability to maintain muscular performance (Latella et al., 2016). Furthermore, an athlete’s psychophysiological state can significantly influence their readiness to perform in subsequent training sessions or competitions, highlighting the need for recovery strategies that address both central and peripheral factors (Kellmann et al., 2018; Rattray et al., 2015).

Transcranial Direct Current Stimulation (tDCS) is a non-invasive brain stimulation technique that delivers weak electrical currents to specific cortical areas to modulate neural activity. Research indicates that tDCS influences corticospinal excitability in a polarity-dependent manner, with anodal stimulation typically increasing and cathodal stimulation decreasing excitability (Dissanayaka et al., 2017). While initially investigated for acute performance enhancement (Machado et al., 2019; da Silva Machado et al., 2021; da Silva Machado and Amiri, 2023), tDCS has more recently been examined as a potential recovery tool due to its neuromodulatory effects on fatigue-related mechanisms (Moreira et al., 2021a; Moreira et al., 2021b; Shiravand et al., 2024; Gonçalves et al., 2024). Studies have demonstrated the recovery benefits of tDCS in athletic populations. Moreira et al. (2021a), Moreira et al. (2021b) reported improved wellbeing and cardiac autonomic control in professional soccer players following post-match tDCS application. Shiravand et al. (2024) found that repeated tDCS sessions enhanced both wellbeing and passing accuracy after simulated matches in male athletes. Gonçalves et al. (2024) further showed that combining tDCS with pneumatic compression over one season yielded superior outcomes for muscle soreness, perceived recovery, and sleep quality compared to compression alone in male soccer players. These collective findings position tDCS as a potentially valuable recovery intervention in sports medicine.

While a limited number of preliminary studies have shown promising results, this research area remains in its early stages, with several critical gaps requiring further investigation, and their results need replication. For instance, all studies agreed that tDCS could improve wellbeing, but whether this improvement translates into better performance remains unknown, as only one study showed improved performance recovery, but in a technical task. There are still doubts about whether tDCS could improve physiological parameters and performance in other exercise/sports modalities, such as endurance exercise. Additionally, the available evidence suggests that the effects of single-session tDCS typically persist for only 60–90 min (Dissanayaka et al., 2017). This duration may limit practical applications in sports settings where pre-competition stimulation is often logistically challenging. Multi-session protocols have shown potential for producing cumulative effects and longer-lasting benefits (Guo et al., 2023; Ke et al., 2023; Ljubisavljevic et al., 2016), though this approach has not been systematically investigated for recovery purposes. Moreover, current research has examined stimulation of either the primary motor cortex (M1) or the dorsolateral prefrontal cortex (DLPFC) separately. However, simultaneous stimulation of the DLPFC and M1 may enhance post-exercise recovery by addressing both neuromuscular and cognitive aspects of fatigue. M1 stimulation increases corticospinal excitability (CSE), while DLPFC stimulation influences effort perception and executive control (Gurdiel-Álvarez et al., 2021; Lattari et al., 2018). Importantly, Vaseghi et al. (2015) demonstrated that concurrent stimulation of these functionally connected regions produced greater and longer-lasting increases in CSE (up to 24 h) compared to single-site stimulation, likely via activation of the DLPFC–premotor–M1 pathway. This dual-site approach has the potential to provide more comprehensive recovery benefits; however, it remains largely unexplored in applied settings. Finally, existing studies on tDCS for post-exercise recovery have predominantly included male participants, with a dearth of information regarding female populations. To date, only a handful of studies have specifically examined females in this context (Moreira et al., 2021a; Lattari et al., 2018). Given documented gender differences in physiological and behavioral responses to tDCS (Bhattacharjee et al., 2022; Rudroff et al., 2020), the generalizability of current findings to female populations requires further investigation. Importantly, the scarcity of research on females is not limited to elite or professional athletes; it also extends to recreationally active women. Studying recreationally active females provides a unique opportunity to explore potential sex-specific responses to tDCS without the confounding effects of highly specialized training regimens. Findings from such studies may help guide both applied sports settings and broader female populations engaging in regular physical activity. These considerations highlight the need for studies examining multi-session protocols, dual-site stimulation approaches, and potential gender-specific effects in athletic recovery contexts.

Building on these considerations and addressing the identified research gaps, this study examined the effects of multi-session, dual-site tDCS (simultaneously targeting the primary motor cortex [M1] and dorsolateral prefrontal cortex [DLPFC]) on post-exercise recovery in females. We evaluated both subjective recovery markers (Wellbeing Questionnaire [WBQ] and Total Quality of Recovery [TQR] scale) and objective performance outcomes (3-km time trial and explosive power measures). We hypothesized that three consecutive days of anodal dual-site tDCS would enhance both subjective recovery metrics and physical performance compared to sham stimulation.

## 2 Methods

### 2.1 Procedure

**FIGURE 1:**
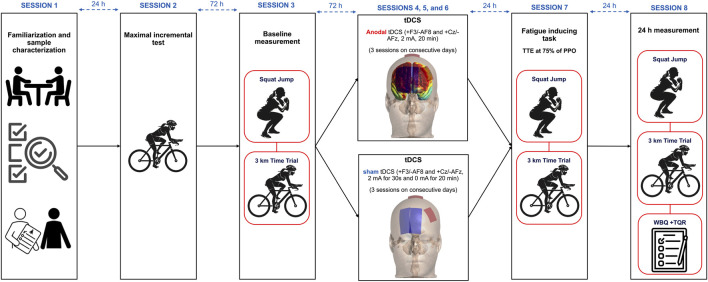
Study design. tDCS = Transcranial Direct Current Stimulation, TTE = Time to Exhaustion test, PPO = peak power output, WBQ = well-being questionnaire, TQR = Total Quality Recovery Scale.

We conducted this randomized, parallel-group, double-blind, sham-controlled trial across eight laboratory sessions. In session 1, we familiarized participants with all procedures and collected participants’ basic data (e.g., personal information, training history, body composition assessment). Participants returned 72 h later for Session 2, where they completed an incremental cycling test to determine their peak power output (PPO). During session 3, participants first performed the Sargent jump test to assess explosive power, followed by a 3-km cycling time trial. From Sessions 4 through 6, we administered either active dual-site anodal tDCS or sham stimulation to participants on three consecutive days, maintaining 24-h intervals between sessions. Twenty-four hours after the final stimulation session (Session 7), participants undertook a time-to-exhaustion (TTE) test at 75% PPO. We recorded their rating of perceived exertion (RPE) and heart rate (HR) at predefined intervals throughout the TTE test. Immediately following this test, participants repeated both the Sargent jump test and 3-km time trial. Session 8 was conducted 24 h later (48 h post-intervention). In this final session, participants completed recovery-related questionnaires and performed their last set of performance assessments, including the Sargent jump test and 3-km cycling time trial. Figure 1 illustrates the complete study design timeline and procedures.

### 2.2 Participants

**FIGURE 2:**
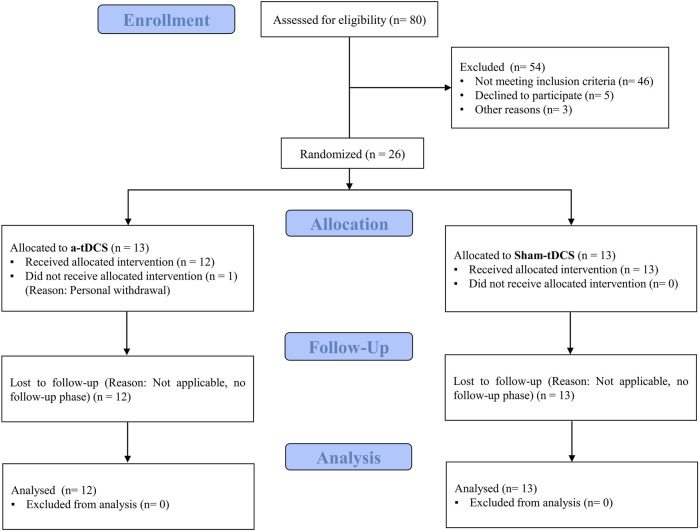
Participants flow.

Twenty-five recreationally active women (aged 18–30 years) voluntarily participated in this study. Participant characteristics are presented in Table 1. The sample size was determined using G*Power software (version 3.1.9.2), based on a repeated-measures ANOVA design (α = 0.05, power = 0.80) for group-by-time interaction (2 groups × 3 measurements). The calculation was informed by the effect size reported in a previous study by Lattari et al. (2018), in which anodal tDCS significantly increased time to exhaustion in physically active women, yielding a medium effect size (Cohen’s d ≈ 0.51). However, to adopt a more conservative approach, we based our sample size calculation on a smaller estimated effect size (f = 0.3). The analysis suggested a minimum of 20 participants; to allow for potential attrition, 26 participants were enrolled. Subjects were randomly allocated to either the dual-site anodal tDCS group (n = 13) or the sham-tDCS group (n = 13). Ultimately, all sham participants and 12 from the anodal tDCS group completed the study protocol. Inclusion criteria comprised: age 18–30 years (to ensure physical capability for the exercise protocols while minimizing age-related variability), at least 3 years of regular, structured endurance-based training at a minimum frequency of three times per week (to ensure a consistent baseline training status), right-handedness (to reduce variability in tDCS response associated with hemispheric lateralization), absence of cardiovascular, pulmonary, or metabolic conditions (necessary given the exhaustive nature of the tests), no history of neurological disorders, no implanted metallic devices in the head (to avoid potential risks and altered current distribution during tDCS), non-smoking status (to avoid potential influences of smoking on cardiovascular and neuromuscular performance), and normal vision (to ensure participants could correctly perceive visual scales during testing). Exclusion criteria included voluntary withdrawal, acute illness or musculoskeletal injury during the study, missed sessions, or inability to complete testing protocols. To control for potential hormonal influences on fatigue and recovery, all participants were tested during the follicular phase of their menstrual cycle (days 7–14), confirmed via self-reported menstrual history. The study was conducted in accordance with the Declaration of Helsinki and approved by the Institutional Ethics Committee (IR.RAZI.REC.1402.048). The trial was registered in the Iranian Clinical Trial Registry (IRCT ID: IRCT20230925059509N1). Data collection was carried out between 25 November 2023, and 15 February 2024, in Kermanshah, Iran. The flow of participants through the study is illustrated in Figure 2.

**TABLE 1:** General characteristics of the participants (n_(Anodal)_ = 12, n_(Sham)_ = 13).

Variables	Mean ± SD (anodal)	Mean ± SD (sham)
Age (years)	22.17 ± 2.08	20.85 ± 2.27
Body Mass (kg)	58.04 ± 7.04	64.59 ± 8.30*
Height (cm)	166.08 ± 5.37	167.38 ± 3.82
Body Mass Index (kg/m2)	21.00 ± 2.00	23.05 ± 2.88
Body Fat (%)	23.34 ± 4.22	27.78 ± 4.96*
Fat Mass (kg)	13.77 ± 3.74	18.27 ± 5.31*
Lean Body Mass (kg)	40.93 ± 3.42	39.81 ± 11.12
Training Sessions (days/week)	3.66 ± 1.15	3.38 ± 0.51
Training Duration (min/day)	105.00 ± 43.38	117.23 ± 71.85
Training Intensity (% HRmax)	73.12 ± 9.66	73.65 ± 12.36
Training Experience (years)	8.87 ± 4.08	9.85 ± 4.71
Peak Power Output (W)	134.49 ± 20.36	132.62 ± 16.05

* = significant difference at baseline.

### 2.3 Randomization, allocation, and concealment

Randomization was performed by an independent researcher not involved in the study. Each participant was first assigned a unique anonymized identification code. Using the website www.randomization.com, this code was entered into a computer-generated randomization list to assign the participant to either the anodal tDCS or sham group. A simple randomization method without blocking was used, and the allocation was stratified based on peak power output (PPO) to ensure a balanced distribution of endurance performance levels across groups. The randomization sequence was accessible only to the independent individual conducting the assignment, thus ensuring allocation concealment and minimizing selection bias. The study followed a double-blind design in which neither the participants nor the principal investigator conducting the outcome assessments were aware of group allocations. To maintain investigator blinding, the lead researcher exited the laboratory during each stimulation session and returned only after the removal of electrodes and sensors. Participant blinding was ensured by placing the stimulation device behind the participant’s head and covering it completely with an opaque cloth, so that no display elements or operational cues were visible throughout the session (Clanton et al., 2012).

### 2.4 Incremental and time to exhaustion tests

In the second session, peak power output (PPO) was assessed using a cycle ergometer (Cyclus 2, RBM Elektronik-automation GmbH, Leipzig, Germany) following the Astrand protocol for women. Participants first completed a standardized 5-min warm-up at a self-selected cadence, then began the incremental exercise test at 50 W with a cadence of 50–60 rpm. The workload was increased by 25 W every 2 min until volitional exhaustion. Exhaustion was defined as the occurrence of at least two of the following criteria: (Kellmann et al., 2018): heart rate ≥90% of age-predicted maximum (220 − age), (Giboin et al., 2018), inability to maintain the cadence (50–60 rpm) for more than 5 s despite verbal encouragement, and (Kargarfard et al., 2018) a rating of perceived exertion (RPE) ≥90 on the 0–100 Borg scale. The power output of the last completed stage was recorded, and PPO was calculated using the following formula: PPO = W_out + (t/120) × 25, where *W_out* is the workload of the last completed stage and *t* is the time (in seconds) sustained at the final stage (Prieto-Bellver et al., 2024; Etemadi et al., 2023). In the seventh session, participants performed a time-to-exhaustion (TTE) cycling task on a cycle ergometer (Cyclus 2, RBM Elektronik-automation GmbH, Leipzig, Germany) to induce physical fatigue. The test began with a 5-min warm-up at 45% of the previously determined PPO, followed by continuous cycling at 75% of PPO with a cadence of 60 rpm until volitional exhaustion. The saddle height was adjusted individually during the second session and was kept identical during this session to ensure consistency. Throughout the task, verbal encouragement was provided to minimize premature termination. Exhaustion was defined by the presence of at least two of the following criteria: (Kellmann et al., 2018): HR ≥ 90% of age-predicted maximal HR (220 − age), (Giboin et al., 2018), inability to maintain the target cadence (60 rpm) for more than 5 s despite encouragement, and (Kargarfard et al., 2018) RPE ≥90 on the 0–100 Borg scale (Etemadi et al., 2023).

### 2.5 Transcranial direct current stimulation (tDCS)

**FIGURE 3:**
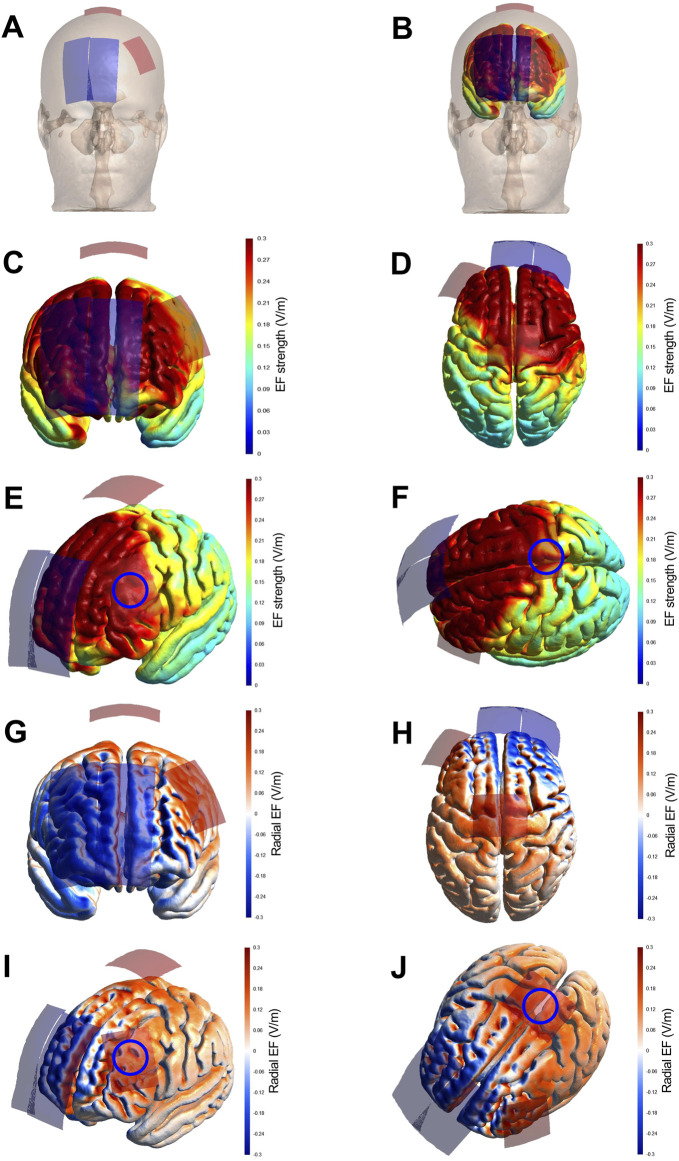
Strength and radial component of the electric field induced by tDCS, reproduced from Banaei et al. (2023). Legend: Finite Element Models derived from Magnetic Resonance Imaging in a head model (MNI152) of the strength and radial (normal to the cortical surface) component of the electric field (EF) induced by tDCS. Electrode montage targeting the simultaneous stimulation with anodal tDCS of the representation of the lower limbs in the primary motor cortex and the left dorsolateral prefrontal cortex **(A,B)**, with red electrodes representing the anodes (5 × 4 cm) and blue electrodes representing the cathodes (9 × 4 cm). The EF strength is presented in the color-coded figures **(C–F)**, with hotter colors indicating stronger EF and colder colors indicating the opposite. The radial EF is presented in the color-coded figures **(G–J)**, where red color represents the electric current flowing into the cortex (i.e., inducing excitatory effects) and blue color represents the electric current flowing out of the cortex (i.e., inducing inhibitory effects). The study montage has reached the target areas with enough electric current strength to induce a neuromodulatory effect, as shown in figures **(E,F)** (blue circles roughly representing the target areas). Furthermore, the target areas were stimulated with the desired polarity (i.e., anodal current) to induce excitatory effects in the target regions, as shown in panels **(I,J)** (blue circles roughly representing the target areas).

Transcranial direct current stimulation (tDCS) was administered daily during sessions 4 to 6 using two battery-driven stimulators (NeuroStim 2, Medina Tebgostar, Tehran, Iran). Stimulation was delivered via four rectangular carbon electrodes encased in saline-soaked sponges (140 mmol NaCl in Milli-Q water): two anodes (5 × 4 cm; 20 cm^2^) and two cathodes (9 × 4 cm; 36 cm^2^). The larger cathodes were selected to minimize their neuromodulatory influence. Target areas were localized using a 64-channel EEG cap aligned with the international 10–20 system. A unihemispheric concurrent dual-site anodal tDCS (a-tDCS_UHCDS) montage was implemented to concurrently stimulate the primary motor cortex (M1) and the left dorsolateral prefrontal cortex (DLPFC) (Gurdiel-Álvarez et al., 2021; Talimkhani et al., 2019). A 2-mA current was applied to each site for 20 min with a ramp-up and down at the beginning and end of the stimulation for 30 s. The first anode was placed 2.5 cm lateral to the midline on either side of Cz, targeting the lower limb representation of M1. The second anode was positioned vertically over F3 to stimulate the left DLPFC. Both cathodes were placed over the supraorbital area, with one centered at AF8 and the other located between Fpz and AFz. This montage was selected based on prior evidence indicating that a-tDCS_UHCDS induces more robust and sustained corticospinal excitability compared to stimulation of M1 or DLPFC alone (Lattari et al., 2018; Talimkhani et al., 2019). The electric field distribution was modeled using a finite element method in SimNIBS 4.0.0 (Thielscher et al., 2015), following established parameters described previously (Banaei et al., 2023). A visual representation of the tDCS-induced current flow is provided in Figure 3. In the sham condition, electrode positioning was identical to that in the anodal condition. During the initial ramp-up for 30 s, the 2 mA current was applied to mimic the initial tingling sensation, followed by a 30-s ramp-down to zero current. The stimulator then remained off for the rest of the session, except for a final 30-s ramp-up and ramp-down sequence at the end to mimic the sensation of stimulation cessation. This protocol has been shown to be effective for blinding participants to the tDCS condition (Moreira et al., 2021a; Moreira et al., 2021b; Etemadi et al., 2023; Banaei et al., 2023; Teymoori et al., 2023).

### 2.6 3-km time trial (TT)

In sessions 3, 7, and 8, participants completed a 3-km self-paced cycling time trial (TT) on a cycle ergometer (Cyclus 2, RBM Elektronik-automation GmbH, Leipzig, Germany). The ergometer was configured with a fixed gear ratio and resistance setting to ensure identical mechanical conditions across trials and participants. The saddle height was individually adjusted during the incremental test in session 2 and replicated for both TT assessments to ensure biomechanical consistency. The first TT was performed at baseline (before tDCS intervention), the second was performed immediately after the time-to-exhaustion (TTE) test in session 7, while the third TT was completed 24 h later during session 8 to assess recovery. In all trials, participants were instructed to complete the 3-km distance as quickly as possible while maintaining a self-selected cadence. No real-time feedback on performance (e.g., distance, elapsed time, or power output) was provided during the task to avoid pacing influence. Standardized verbal encouragement was given throughout the trial to motivate maximal effort. Performance was quantified as the total time (in seconds).

### 2.7 Explosive power (Sargent’s jump test)

Vertical jump performance was assessed using a wall-based reach-and-jump method. Participants stood adjacent to a wall marked in centimeters and extended their dominant arm upward while keeping both feet flat on the ground; the highest point reached by the middle fingertip was recorded as the standing reach height. Subsequently, they performed Sargent’s jump test using both legs, aiming to touch the wall at the peak of their jump. The highest point reached during the jump was recorded as the jump height. Each participant completed two trials, and the best attempt was used for analysis. Explosive lower-limb power was estimated by calculating vertical displacement (i.e., jump height minus standing reach height). Additionally, a power efficiency index was calculated using the following formula: Efficiency Index = [Body weight (lb) × Jump height (inch)]/Standing height (inch) (Clanton et al., 2012; de Salles et al., 2012).

### 2.8 Wellbeing Questionnaire (WBQ) and Total Quality recovery scale (TQR)

Subjective recovery status was evaluated using the Total Quality Recovery (TQR) scale (Moreira et al., 2021b), a single-item measure ranging from 6 (“very, very poor recovery”) to 20 (“very, very good recovery”), with higher scores indicating better perceived recovery. In addition, the Wellbeing Questionnaire (WBQ) (Moreira et al., 2021a) was used to assess five dimensions of recovery-related wellbeing: fatigue, sleep quality, muscle soreness, stress, and mood. Each item was rated on a 5-point Likert scale, and the total WBQ score ranged from 5 to 25, with lower scores reflecting poorer overall wellbeing and higher scores indicating better status (Moreira et al., 2021a; Moreira et al., 2021b; Shiravand et al., 2024). Participants were familiarized with the format and response procedures for both questionnaires during the familiarization session. Both the TQR and WBQ assessments were administered in the final experimental session (session 8), which took place 24 h after the endurance fatiguing protocol.

### 2.9 Statistical analysis

Data are reported as mean ± standard deviation (M ± SD). The Shapiro–Wilk test was used to assess normality assumptions. To analyze changes in explosive power (Sargent’s jump test) and 3-km time trial performance across time and groups, a mixed-model ANOVA with repeated measures (2 × 3 factorial design: group × time) was conducted. In cases of significant group × time interaction effects, follow-up analyses included (Kellmann et al., 2018) repeated-measures ANOVA within each group to examine time effects, with Bonferroni-corrected pairwise comparisons, and (Giboin et al., 2018) independent-samples t-tests between groups at each time point, also corrected using Bonferroni adjustment. Group comparisons for single-timepoint variables (HR, RPE, TQR, WBQ) were performed using independent-samples t-tests. When the assumption of sphericity was violated, Greenhouse–Geisser corrections were applied. For non-normally distributed variables, Friedman tests were used for within-group time comparisons, and Mann–Whitney U tests were employed for between-group comparisons, with Bonferroni-adjusted *post hoc* tests applied as necessary. Effect sizes were reported as partial eta-squared (η^2^_p_) for ANOVA outcomes, interpreted as small (0.01–0.059), moderate (0.06–0.139), or large (≥0.14), and Cohen’s *d* for pairwise comparisons, categorized as small (0.20–0.49), medium (0.50–0.79), or large (≥0.80). All statistical analyses were performed using SPSS version 27 (SPSS Inc., Chicago, IL, United States of America), with the significance level set at *p* < 0.05.

## 3 Results

The overall results of the study are presented in Tables 2, 3. A total of 12 participants in the anodal tDCS group and 13 participants in the sham group completed all study procedures and were included in the final statistical analysis. At baseline, no significant differences were observed between the tDCS and sham groups for the characteristics listed in Table 1, except for body mass, fat percentage, and fat mass, which were significantly higher in the tDCS group.

**TABLE 2:** Mean value of the perceived recovery (TQR) and well-being (WBQ) in two different stimulation conditions (n Anodal = 12, n Sham = 13)..

Experimental conditions		
Variables	Anodal Stimulation Group	Sham Stimulation Group
TQR _(6–20 Scale)_	18.25 ± 1.76	16.77 ± 2.35
WBQ _(1–5 Likert Type)_	10.42 ± 4.05	11.54 ± 2.44

Note: TQR = total quality recovery, WBQ = Wellbeing Questionnaire.

**TABLE 3:** Mean value of the lower-limb explosive performance (SJT) and endurance performance (3 km TT) under two different stimulation conditions at specified time points (n Anodal = 12, n Sham = 13).

Experimental conditions						
Variables	Anodal stimulation group			Sham stimulation group		
	Baseline	Post-24	Post-48	Baseline	Post-24	Post-48
SJT _(IF)_	30.09 ± 2.57	27.25 ± 3.04	29.91 ± 2.20	31.99 ± 5.80	29.80 ± 5.28	30.56 ± 6.26
3 km TT _(min)_	03:33.38 ± 00:24.16	03:28.03 ± 00:23.81	03:09.35 ± 00:17.12	03:34.35 ± 00:26.54	03:37.68 ± 00:23.04	03:25.40 ± 00:25.66

Note: SJT = Sargent Jump Test, 3 km TT = 3 km time trial.

### 3.1 Heart rate and RPE during the time to exhaustion test

**FIGURE 4:**
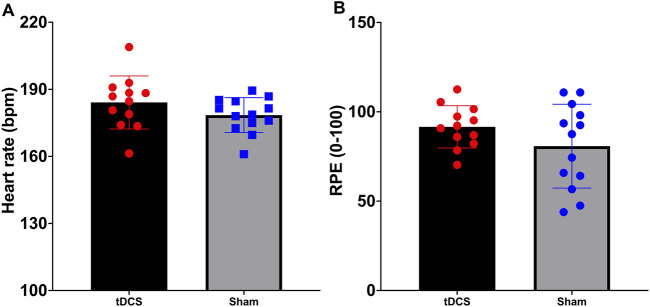
Maximum heart rate **(A)** and ratings of perceived exertion **(B)** during the time to exhaustion test to induce fatigue.

To compare heart rate (HR) and rating of perceived exertion (RPE) during the exhaustive endurance task between the anodal tDCS group and the sham tDCS group, independent samples t-tests were conducted. For heart rate, Levene’s test indicated that the assumption of equal variances was met (*F*(1,22) = 1.177, *p* = 0.289), and therefore the standard t-test was applied. The difference between the two groups was not statistically significant (*t*(22) = 1.422, *p* = 0.169, d = 0.56), with a mean difference of 5.67 bpm (95% CI: 2.58–13.91). For perceived exertion, Levene’s test indicated unequal variances (*F*(1,22) = 10.267, *p* = 0.004), so the unequal variance t-test was used. Again, no significant difference was observed between the groups (*t*_(18.038)_ = 1.469, *p* = 0.159, d = 0.57), with a mean difference of 10.79 units (95% CI: 4.64–26.23). These findings indicate that both groups experienced comparable exercise intensity, as reflected by similar heart rate responses and perceived exertion levels during the endurance task (Figure 4).

### 3.2 3-km time trial (TT)

**FIGURE 5:**
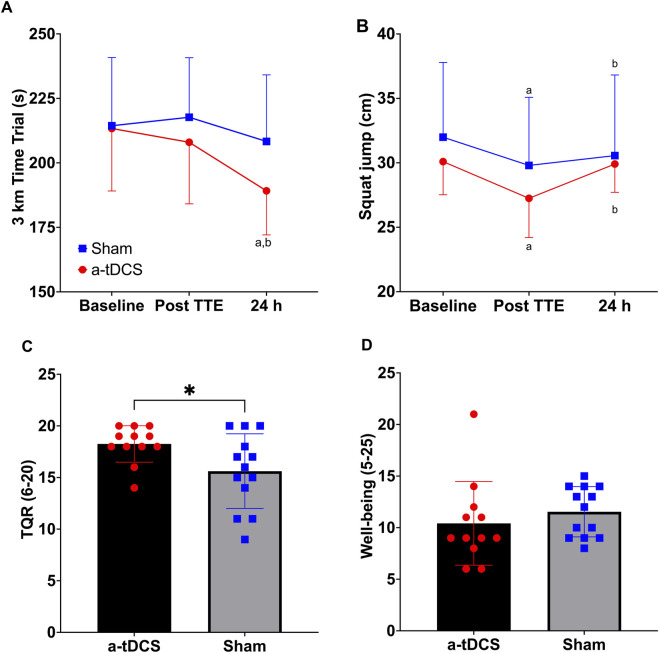
Three kilometers Time trial **(A)** and squat jump **(B)** at baseline, after the fatiguing protocol (TTE: time to exhaustion test) and at 24 h of recovery in the anodal and sham Transcranial Direct Current Stimulation groups. Total Quality Recovery **(C)** and wellbeing **(D)** at 24 h of recovery. ^a^ = significantly different from baseline (all ps < 0.01); ^b^ = significantly different from post TTE (all ps < 0.01); * = significantly different from sham (p = 0.046).

Statistical analysis revealed a significant group × time interaction (F (2, 46) = 4.55, p = 0.036, η^2^_p_ = 0.134) and main effect of time (F (2, 46) = 12.24, p < 0.001, η^2^_p_ = 0.347) for the 3-km time trial performance. No significant main effect of group was observed (F (1, 23) = 1.31, p = 0.26, η^2^_p_ = 0.054). Post-hoc analysis demonstrated that the anodal tDCS group showed significantly improved performance at 24 h post-intervention compared to both baseline (p < 0.001, d = 1.54, 95% CI [-36.71, −11.33]) and post-TTE measurements (p < 0.001, d = 1.56, 95% CI [-28.4, −8.96]). However, between-group comparisons at baseline and post-TTE were non-significant (*p* > 0.05). At 24 h post-intervention, the raw *p*-value for the between-group comparison was 0.03; however, this did not remain significant after Bonferroni correction for multiple comparisons (adjusted α = 0.0167) and was therefore considered non-significant (Figure 5A).

### 3.3 Explosive power (Sargent’s jump test)

A significant main effect of time was observed for Sargent’s jump test (SJT) performance (F (2, 46) = 8.93, p = 0.001, η^2^_p_ = 0.280), reflecting changes across the three time points. No significant effects were found for group (F (1, 23) = 1.02, p = 0.324, η^2^_p_ = 0.042) or the group × time interaction (F (2, 46) = 1.26, p = 0.295, η^2^_p_ = 0.052). Post-hoc Bonferroni-corrected comparisons indicated a significant decline in SJT performance from baseline to post-TTE (Δ = 2.52, p = 0.005, 95%CI [0.686, 4.351]), followed by a recovery at 24 h (Δ = 1.71, p = 0.009, 95%CI [0.383, 3.042]). Baseline and 24-h performance did not differ significantly (p > 0.05; Figure 5B).

### 3.4 Wellbeing Questionnaire (WBQ) and Total Quality recovery (TQR) scale

Independent-samples Mann–Whitney U tests showed that TQR scores at 48 h post-intervention were significantly higher in the tDCS group compared with the sham group [(U = 41.50, z = −2.06, p = 0.046 (two-tailed, exact), 95% CI [0.000, 2.000], d = −0.41; Figure 5C)]. In contrast, there was no statistically significant difference between the groups for WBQ scores [(U = 103.50, z = 1.41, p = 0.168 (two-tailed, exact), 95% CI [-4.000, 1.000], d = 0.28; Figure 5D)].

## 4 Discussion

The main findings of this study revealed that while the a-tDCS group reported significantly greater perceived recovery (TQR scores), we observed no between-group differences in wellbeing (WBQ), 3-km time trial performance, or explosive power (Sargent jump test). These results suggest that a short-term, multi-session dual-site a-tDCS protocol may enhance subjective recovery perception without significantly affecting performance recovery or overall wellbeing. To our knowledge, this represents the first investigation of three consecutive dual-site a-tDCS sessions (targeting both M1 and L-DLPFC simultaneously) on subjective and performance recovery metrics in recreationally active females following submaximal endurance exercise. Our protocol specifically examined the effects after a time-to-exhaustion test at 75% peak power output, providing novel insights into tDCS applications for endurance recovery.

While central mechanisms of fatigue are well-established contributors to performance decline (Taylor et al., 2016; Taylor and Gandevia, 2008; Gandevia, 2001), recovery strategies specifically targeting the central nervous system, particularly the brain, remain understudied (Rattray et al., 2015; Minett and Duffield, 2014). Our findings demonstrate that tDCS enhanced perceived recovery without significantly affecting wellbeing, contrasting with previous reports (Moreira et al., 2021a; Moreira et al., 2021b). One possible explanation for this dissociation is that stimulation of the DLPFC may preferentially influence cognitive and perceptual aspects of recovery, such as effort regulation, motivation, and interoceptive awareness, rather than directly improving neuromuscular or physiological recovery processes. This is consistent with evidence showing that DLPFC activation can modulate perceived exertion and fatigue without necessarily altering physical output (Robertson and Marino, 1985; Holgado et al., 2019). In our study, this could have led to higher TQR scores in the tDCS group despite the absence of measurable performance benefits. Conversely, the lack of performance improvement may reflect stimulation parameters (e.g., duration, intensity, montage) or the possibility that both groups achieved near-complete recovery within 24 h, leaving limited scope for additional enhancement. Another factor that may have contributed to the higher TQR scores in the tDCS group is the potential influence of placebo or expectancy effects. Even under rigorous double-blind conditions, participants receiving active stimulation might perceive that they are benefiting from an “active” recovery aid, which could positively bias their subjective ratings. Although our sham protocol was designed to mimic the sensory experience of active tDCS closely and has been validated as effective for maintaining participant blinding (Ambrus et al., 2012), placebo-related mechanisms cannot be completely excluded as a confounding factor.

In the context, Moreira et al. (2021a), Moreira et al. (2021b) found improved wellbeing and cardiac autonomic control, but not perceived recovery in professional soccer players following an official match with a single session of tDCS application. The authors attributed these benefits to potential stimulation effects on the anterior insula (involved in emotional regulation and interoception) through bilateral DLPFC stimulation, while suggesting that this may enhance subjective recovery. High inter-individual variability might explain the null TQR findings (Moreira et al., 2021a; Moreira et al., 2021b). Shiravand et al. (2024) similarly reported that three DLPFC-targeted tDCS sessions improved both wellbeing and passing accuracy in youth soccer players after match simulation. The discrepancies in subjective recovery measures across studies may stem from several factors: (Kellmann et al., 2018): stimulation targets (isolated DLPFC vs. our combined DLPFC + M1 approach); (Giboin et al., 2018); number of tDCS sessions (1 vs. 3 sessions); (Kargarfard et al., 2018); participant characteristics (only one prior recovery study included female individuals); and (Baghaei et al., 2022) fatigue protocols (90+ minute soccer matches vs. our <45 min time-to-exhaustion test).

A novel aspect of this study was the inclusion of performance-based recovery measures to evaluate whether tDCS affects physical and physiological parameters beyond subjective measures. Our results indicate that three sessions of concurrent dual-site tDCS did not improve either explosive performance (assessed via squat jump) or 3-km time trial performance, suggesting this specific stimulation protocol does not influence these athletic performance metrics. Previous research on tDCS and post-exercise recovery has primarily examined subjective recovery measures (e.g., WBQ, TQR) or physiological parameters (e.g., heart rate variability), with only one study assessing countermovement jump and sport-specific performance (Shiravand et al., 2024). Our findings regarding explosive lower limb performance align with those of Shiravand et al. (2024), confirming no tDCS effect on this physical capacity. The current study extends these observations by demonstrating that cycling performance similarly remains unaffected, thereby broadening our understanding of tDCS effects on performance recovery.

Notably, while the 3-km time trial performance in the tDCS group did not differ significantly from the sham group, performance in the tDCS group declined over time relative to both baseline and post-TTE measurements, whereas the sham group maintained stable performance. These findings suggest the need for future studies to extend the post-fatigue evaluation period beyond 24 h. Our results partially align with Holgado et al. (2019), who reported no significant effects of single-session DLPFC-targeted tDCS on self-paced time trial performance in trained cyclists, although their study did not specifically assess recovery. The current literature presents mixed findings regarding tDCS effects on explosive performance. Grospretre et al. (2021) found that while DLPFC-targeted tDCS had no effect on vertical jump performance, extracephalic M1 stimulation improved jump outcomes in parkour athletes, potentially through combined supraspinal and spinal neuromodulation. Similarly, Shiravand et al. (2024) observed no effect of three DLPFC-targeted tDCS sessions on countermovement jump performance following soccer match simulation, though it should be noted that their fatigue protocol itself did not affect countermovement jump performance. This raises questions about either the sensitivity of this test for detecting neuromuscular fatigue or the fatigue-inducing capacity of their protocol. In contrast, our study demonstrated that squat jump performance decreased post-TTE in both groups, confirming successful fatigue induction. However, we found no effect of tDCS on squat jump recovery.

While our study employed three sessions of concurrent M1 and DLPFC anodal tDCS to induce lasting effects, both the number of sessions and measurement timeline may have been insufficient to demonstrate tDCS-mediated recovery benefits. Recent work by Goncalves et al. (Gonçalves et al., 2024) provides relevant insights: their season-long intervention combining DLPFC-targeted tDCS with pneumatic compression (applied post-match) significantly improved pain perception, sleep quality, subjective recovery, and creatine kinase levels in elite Brazilian soccer players compared to compression alone. The authors attributed these benefits to synergistic effects on central and peripheral recovery mechanisms (Gonçalves et al., 2024). These findings suggest that more extensive tDCS protocols (greater number of sessions) combined with complementary recovery modalities may yield more robust recovery enhancements.

## 5 Limitations and future directions

While this study maintained rigorous experimental controls, several limitations should be considered when interpreting the results. The relatively small sample size and exclusive inclusion of trained female participants address a clear gap in the literature but limit the generalizability of the findings to other populations, such as male athletes or less-trained individuals. Additionally, the short-term nature of the intervention means that longer-term or repeated use of tDCS was not examined, preventing conclusions regarding potential cumulative or sustained effects. The absence of neuroimaging (e.g., EEG or fMRI) precludes mechanistic insights into potential neural changes associated with tDCS. Furthermore, the lack of long-term follow-up assessments limits our understanding of whether the observed effects persist beyond the study period. Future investigations should address these limitations by recruiting larger and more diverse samples, implementing extended intervention protocols, and incorporating multimodal assessment tools.

## 6 Conclusion

This study demonstrates that three consecutive sessions of dual-site anodal tDCS (targeting both M1 and L-DLPFC) may improve perceived recovery following submaximal endurance exercise, without measurable effects on wellbeing, lower-limb explosive power, or 3-km cycling time trial performance. These findings suggest that this stimulation protocol could be considered in scenarios with compressed training or competition schedules and limited recovery time; however, coaches and practitioners should note that the benefit appears to be subjective only and does not confer any measurable performance advantage.

## Data Availability

The raw data supporting the conclusions of this article will be made available by the authors, on reasonable request and without undue reservation.

## Abbreviations

CNS, Central Nervous System; DLPFC, Dorsolateral Prefrontal Cortex; EF, Electric Field; EI, Efficiency Index; HR, Heart Rate; M1, Primary Motor Cortex; PPO, Peak Power Output; RPE, Rating of Perceived Exertion; SJT, Sargent Jump Test; TT, 3-Km Time Trial; TQR, Total Quality Recovery Scale; tDCS, transcranial Direct Current Stimulation; TTE, Time to Exhaustion; WBQ, Well-Bing Questionnaire; W, Watt.
